# Effect of the COVID-19 pandemic on Tennessee hospital antibiotic use

**DOI:** 10.1017/ash.2022.90

**Published:** 2022-05-16

**Authors:** Youssoufou Ouedraogo, Christopher Evans, Daniel Muleta, Christopher Wilson

## Abstract

**Background:** On March 5, 2020, the Tennessee Department of Health (TDH) announced the first case of COVID-19 in the state. Since then, hospitals have been overwhelmed by the spike in respiratory infections. Several studies have attempted to describe the impact of the pandemic on antibiotic prescriptions. The NHSN Antimicrobial Use Option offers a platform for hospitals to report their antibiotic usage. The TDH has established access to hospital antibiotic usage data statewide through an existing NHSN user group. We compared the change in the volume of inpatient antibiotic prescriptions before and during the pandemic. **Methods:** An ecological study was conducted from January 2019 to December 2021. Aggregated facility-level data from the NHSN Antimicrobial Use Option were used to describe antibacterial use among Tennessee hospitals. Data from facilities that had reported at least 1 month of data during the study period were included in this study. The antimicrobial use rate was calculated by dividing the antimicrobial days of therapy (DOT) by the number of 1,000 days present. Overall antimicrobial use rates as well as specific antimicrobial use rates for azithromycin, ceftriaxone, and piperacillin–tazobactam were compared across years. **Results:** In total, 55 hospitals reported at least 1 month of data into the NHSN Antimicrobial Use Option during the study period. These hospitals had a median bed size of 140 (range, 12–689). **Conclusions:** We observed a modest increase in overall antibiotic use during the COVID-19 pandemic in Tennessee facilities. This trend appeared to be primarily attributed to agents used for community-acquired respiratory infections, such as azithromycin and ceftriaxone, earlier in the pandemic. However, both of these agents have fallen to prepandemic use levels during 2021. The fact that overall use increased in 2021 suggests that other agents not analyzed may have contributed to this effect. Further analysis may help determine which agents are responsible for this increase in 2021.

**Funding:** None

**Disclosures:** None

